# A Compact Non-Ionizing RF Bioelectronic Sensor for Translational Phantom-Based Detection of Tumor-Like Dielectric Changes in Bone

**DOI:** 10.7150/ntno.137930

**Published:** 2026-08-12

**Authors:** Tarlan Mahouti, Hakan Yilmazer, Ladislau Matekovits, Hakan Eroğlu, Mehmet Ali Belen

**Affiliations:** 1 Department of Metallurgical and Materials Engineering, Yildiz Technical University, Istanbul, Turkey.; 2 Department of Electronics and Telecommunications, Politecnico di Torino, Turin, Italy.; 3 Politehnica University Timisoara, 300223 Timisoara, Romania.; 4 Instituto di Elettronica e di Ingegneria dell'Informazione e delle Telecomunicazioni, National Research Council of Italy, 10129 Turin, Italy.; 5 Department of Electric and Electronical Engineering, İskenderun Technical University, Hatay, Turkey.

**Keywords:** RF bioelectronic sensor, microwave biosensing, bone tumor phantom, dielectric characterization, translational diagnostics, reflection coefficient, resonance frequency shift, S_11_ measurement

## Abstract

**Introduction:**

Early identification of tumor-like changes in bone remains a major diagnostic challenge because conventional imaging methods mainly rely on structural contrast and may involve ionizing radiation, high cost, or limited point-of-care accessibility. In line with the growing interest in bioelectronic and nanoelectronic biosensor technologies for translational diagnostics, this study investigates a low-cost, non-ionizing microwave sensing platform for detecting dielectric changes associated with a tumor-mimicking bone phantom.

**Methods:**

A compact microstrip resonator antenna was designed in CST Microwave Suite and fabricated on an FR4 substrate for localized near-field sensing. A multilayer bone phantom containing cortical bone- and marrow-mimicking regions was prepared using wheat flour, deionized water, dextrose, and olive oil. A separate water-rich gelatin-based tumor phantom was prepared to reproduce the higher dielectric response expected from malignant tissue. The dielectric properties of the bone and tumor phantoms were measured using a Vector Network Analyzer (VNA)-based open-ended coaxial probe system. The fabricated antenna was then experimentally evaluated by reflection coefficient (S_11_) measurements at two healthy phantom positions and one tumor-over-phantom position.

**Results:**

Dielectric characterization confirmed a clear contrast between the bone phantom and tumor-like region in both the real and imaginary parts of relative permittivity. The tumor-loaded configuration produced a downward resonance shift of 110 MHz around 4.2-4.3 GHz, relative to the healthy reference, which was 2.75 times larger than the variation between the two healthy positions. In addition, a 4.47 dB change in S_11_ magnitude was observed, confirming that the tumor-like inclusion altered the near-field dielectric loading and impedance-matching condition of the resonator.

**Conclusions:**

The proposed microstrip resonator antenna demonstrates the feasibility of a compact RF bioelectronic sensing approach for detecting tumor-like dielectric perturbations in a controlled bone phantom environment. Although not intended as a clinical diagnostic device at this stage, the results support further development of this platform toward translational microwave biosensing, more realistic preclinical phantoms, array-based configurations, and AI-assisted classification for future diagnostic applications.

## Introduction

Bone cancer includes malignant diseases that affect the skeletal system, such as primary tumors including osteosarcoma and chondrosarcoma, as well as secondary metastatic lesions from breast, prostate, and lung cancers 1. These diseases can damage bone structure and strongly affect patient outcomes, especially when detection is delayed 2. Therefore, early identification of abnormal changes in bone tissue remains an important goal in clinical oncology 3.

Conventional imaging methods, including X-ray radiography, computed tomography (CT), and magnetic resonance imaging (MRI), are widely used for bone tumor evaluation. X-ray imaging is fast and useful for detecting visible bone damage, cortical erosion, and mineral changes 4. CT provides three-dimensional information about cortical bone and mineralized tissue, which is helpful for surgical planning 5. MRI is useful for evaluating bone marrow and soft-tissue involvement because of its high soft-tissue contrast 4. However, X-ray and CT use ionizing radiation, which can limit repeated use, especially in children and long-term follow-up cases 6. MRI does not use ionizing radiation, but cortical bone is difficult to image because of its low proton density and very short relaxation time 7. These limitations support the need for complementary, low-cost, and non-ionizing sensing methods.

Microwave sensing is a promising complementary method because it depends on tissue dielectric properties rather than only on structural images 8,9. The main sensing mechanism is based on dielectric contrast between healthy and abnormal tissue regions. This contrast is related to differences in water content, ion concentration, cell structure, membrane state, and metabolic activity 9-11. In the microwave range, these differences appear as changes in complex relative permittivity, including the real part of permittivity (ε′) and the imaginary part of permittivity (ε″) 9,12. Healthy bone tissues, such as cortical bone and lipid-rich marrow, usually have lower dielectric values because of their low water content and high lipid fraction 8,13. In contrast, tumor-like regions may show higher ε′ and ε″ values because they often contain more water and ionic content than surrounding bone tissue 10,13.

Microstrip resonator antennas are suitable for localized near-field microwave sensing because they are planar, compact, low-cost, and easy to connect with standard radiofrequency measurement systems 10,12. Their sensing principle depends on the change in resonant frequency caused by the effective permittivity around the resonator 14. When a material with different dielectric properties is placed in the near-field region, the resonant condition changes. As a result, the reflection coefficient (S_11_) shows a resonance frequency shift and a change in the depth or shape of the resonance notch 10. This makes the microstrip resonator antenna a simple one-port sensing element for detecting dielectric changes in phantom-based studies 12,14.

Phantom-based testing is an important step in the development of microwave biomedical sensing systems 15. Tissue-mimicking phantoms allow the sensor response to be tested under controlled and repeatable conditions that approximate real tissue dielectric behavior 16. For bone tumor sensing, a useful phantom should represent the low-dielectric behavior of cortical bone and marrow, together with the higher dielectric response of a tumor-like region 13. Such controlled experiments provide a bridge between simulation and future ex vivo or in vivo studies, while reducing the ethical, practical, and biological variability problems related to real tissue measurements 15-17.

The main contributions of this study are as follows; First, a compact microstrip resonator antenna was designed on an FR4 substrate and optimized in CST Microwave Studio to obtain a resonant response near 4.0-4.5 GHz for near-field phantom sensing. Second, a multilayer bone phantom, including cortical bone and marrow-mimicking layers, was prepared together with a separate tumor-region phantom using defined material formulations. Third, the dielectric properties of the fabricated bone phantom and tumor region were measured as frequency-dependent ε′ and ε″ values. Fourth, S_11_ measurements were performed at healthy and tumor-over-phantom positions to show a detectable resonance shift and S_11_ change caused by near-field dielectric loading.

Recent studies in nanotheranostics have shown that cancer detection is increasingly moving toward diagnostic platforms that convert tumor-associated biological, molecular, optical, magnetic, or bioelectrical changes into measurable signals 18-20. Examples include SERS (Raman scattering) based deep Raman detection of solid tumors through heterogeneous tissues 18, magnetic particle spectroscopy combined with nanotheranostic platforms for cancer-related circulating tumor cell detection 19, and electrostatic nanoparticle-based capture of tumor cells through cancer-associated surface charge differences 20. Although the present study does not use a nano- or molecular theranostic system, it follows a similar signal-conversion principle. Here, the tumor-like contrast is based on dielectric differences, and this contrast is converted into an RF response through measurable S_11_ resonance changes using a compact microstrip resonator antenna.

In this context, the present study introduces a compact RF bioelectronic sensing platform for phantom-based detection of tumor-like dielectric perturbations in bone-mimicking media. Different from studies that only report antenna performance in free space or simple homogeneous phantoms, this work combines multilayer tissue-mimicking phantom preparation, dielectric characterization, near-field microstrip resonator design, and experimental S_11_-based validation. This multidisciplinary workflow is relevant to translational diagnostic research and biosensor-oriented sensing platforms, where local tissue-related dielectric changes are converted into measurable electromagnetic signatures.

## Materials and Methods

### Microstrip Resonator Antenna Design

The sensing element of the proposed system is a microstrip resonator antenna designed in CST Microwave Studio. The antenna was designed on an FR4 substrate with a relative permittivity of ε_r_ = 4.6 and a thickness of h = 1.56 mm. The metallic resonator and feed line were placed on the upper surface of the substrate, while a ground plane was placed on the opposite surface. The same substrate thickness and relative permittivity were used in both the numerical model and the fabricated prototype.

The electrical properties of the FR4 substrate directly affect the antenna response. The relative permittivity controls the guided wavelength and therefore influences the physical dimensions required to obtain resonance in the selected frequency range. The substrate thickness affects the confinement and extension of the fringing electric field above the antenna surface. These fringing fields are important because they form the main interaction region between the resonator and the material under test. The dielectric loss of FR4 also affects the quality factor, bandwidth, and depth of the S_11_ resonance. Although FR4 has a higher dielectric loss than low-loss microwave laminates, it was selected because of its low cost, mechanical stability, wide availability, and compatibility with standard printed circuit board fabrication.

**Figure 1:**
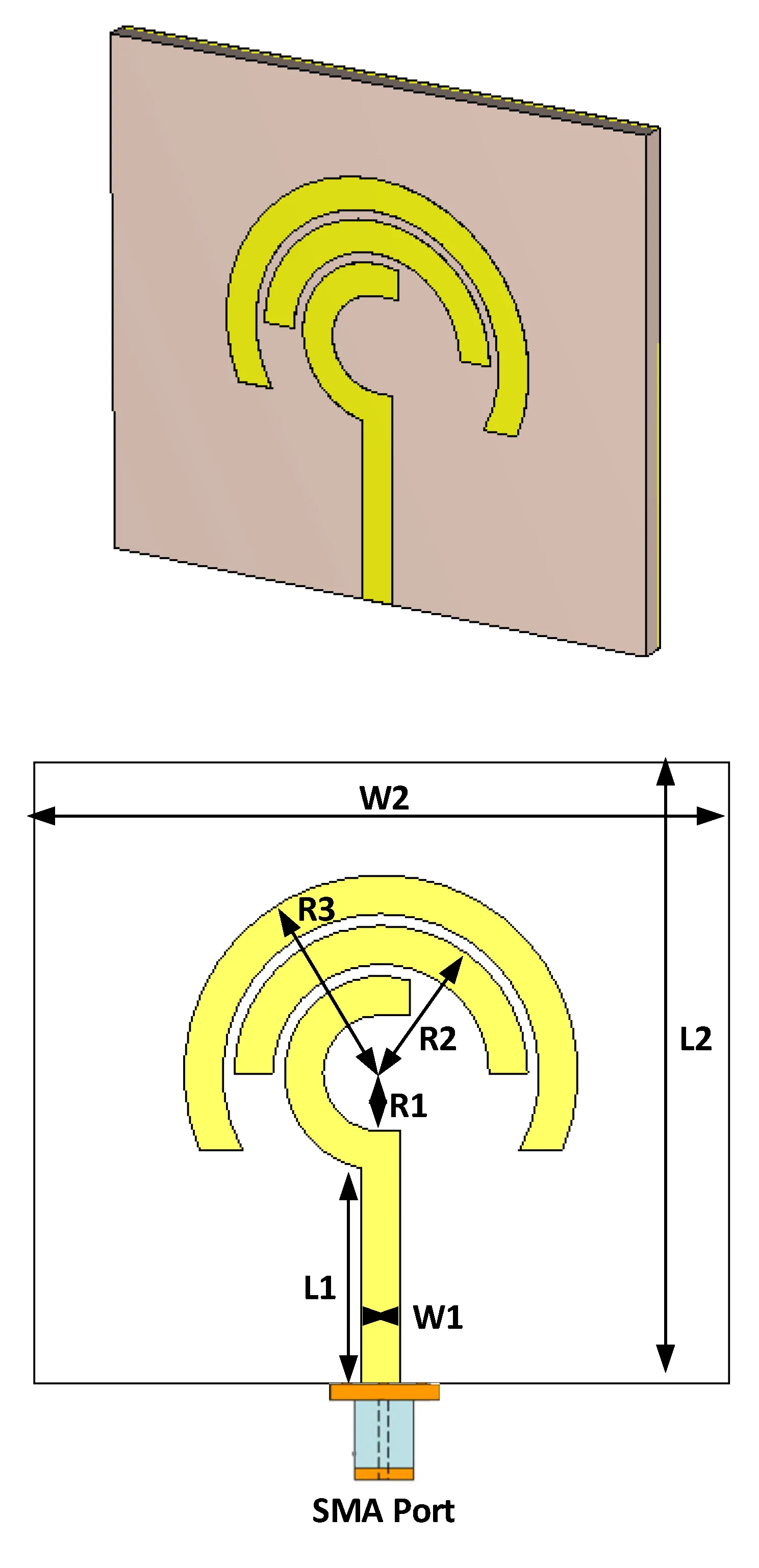
Proposed microstrip resonator antenna geometry as modeled in CST Microwave Studio, showing the overall planar layout on FR4 substrate with the defined geometric features.

The antenna geometry consists of linear and curved resonator sections designed to provide both a clear resonant response and sufficient near-field interaction with the sensing medium. The geometry was optimized through parametric simulations to obtain a well-defined S_11_ resonance notch within the target frequency range of 4.0-4.5 GHz. During this process, the feed-line dimensions, main resonator dimensions, and curved-edge radii were varied to examine their effects on the resonance frequency, impedance matching, and electric-field distribution. The final CST model of the proposed antenna is shown in Fig. 1, and the optimized geometrical parameters are listed in Table 1.

The parameters L1 and W1 define the length and width of the microstrip feed section, respectively. These parameters control the electromagnetic coupling between the 50-Ω measurement port and the resonator. Changes in the feed dimensions can modify the input impedance and therefore affect the depth and shape of the S_11_ resonance notch.

The parameters L2 and W2 define the main dimensions of the resonating structure. L2 mainly controls the effective electrical length and has a strong effect on the resonance frequency. W2 affects the transverse resonator area, capacitive loading, and spatial extent of the electric field above the antenna surface. Together, these parameters determine the main operating frequency range and the effective sensing area of the resonator.

The parameters R1, R2, and R3 define the curved sections along the resonator boundary. These curved sections modify the surface-current path and redistribute the fringing electric field near the antenna edges. During the parametric optimization, the radii were adjusted to obtain a clear resonance notch and to improve electric-field concentration close to the sensing surface. The final parameter values presented in Table 1 represent the optimized geometry used for both simulation and fabrication.

From a sensing point of view, the antenna works as a near-field resonator. Its resonant frequency is affected by the effective permittivity around the fringing electric fields. When a dielectric material is placed close to the resonator, the effective permittivity changes and the resonance frequency shifts. A higher local ε′ increases electric energy storage and generally shifts the resonance to a lower frequency. A higher ε″ increases dielectric loss and changes the depth and bandwidth of the S_11_ notch. Therefore, the antenna response is sensitive to both permittivity and loss changes in the near-field region.

### Electric Field Behavior

**Figure 2:**
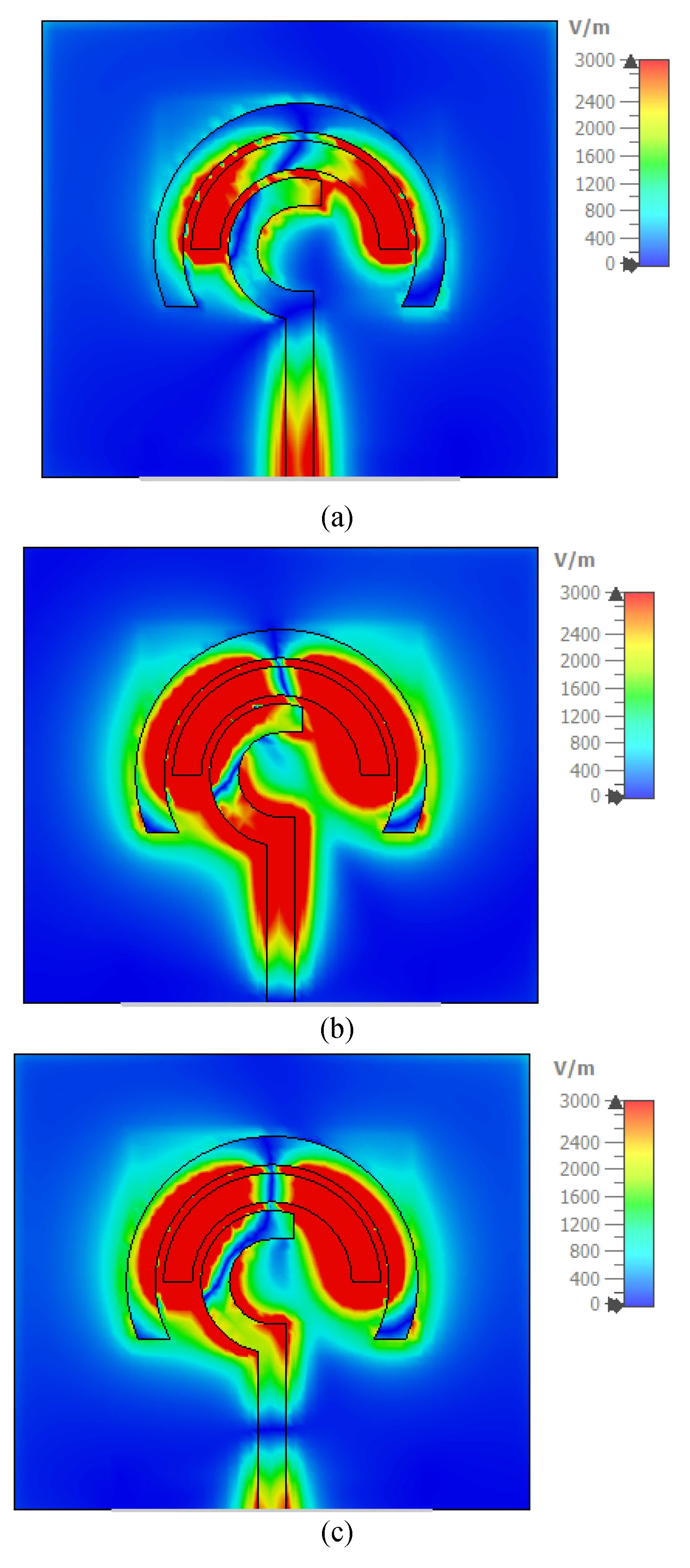
Simulated electric-field distribution of the proposed microstrip resonator antenna at 3.5 GHz: (a) 0° plane, (b) 90° plane, and (c) 145° plane.

**Figure 3:**
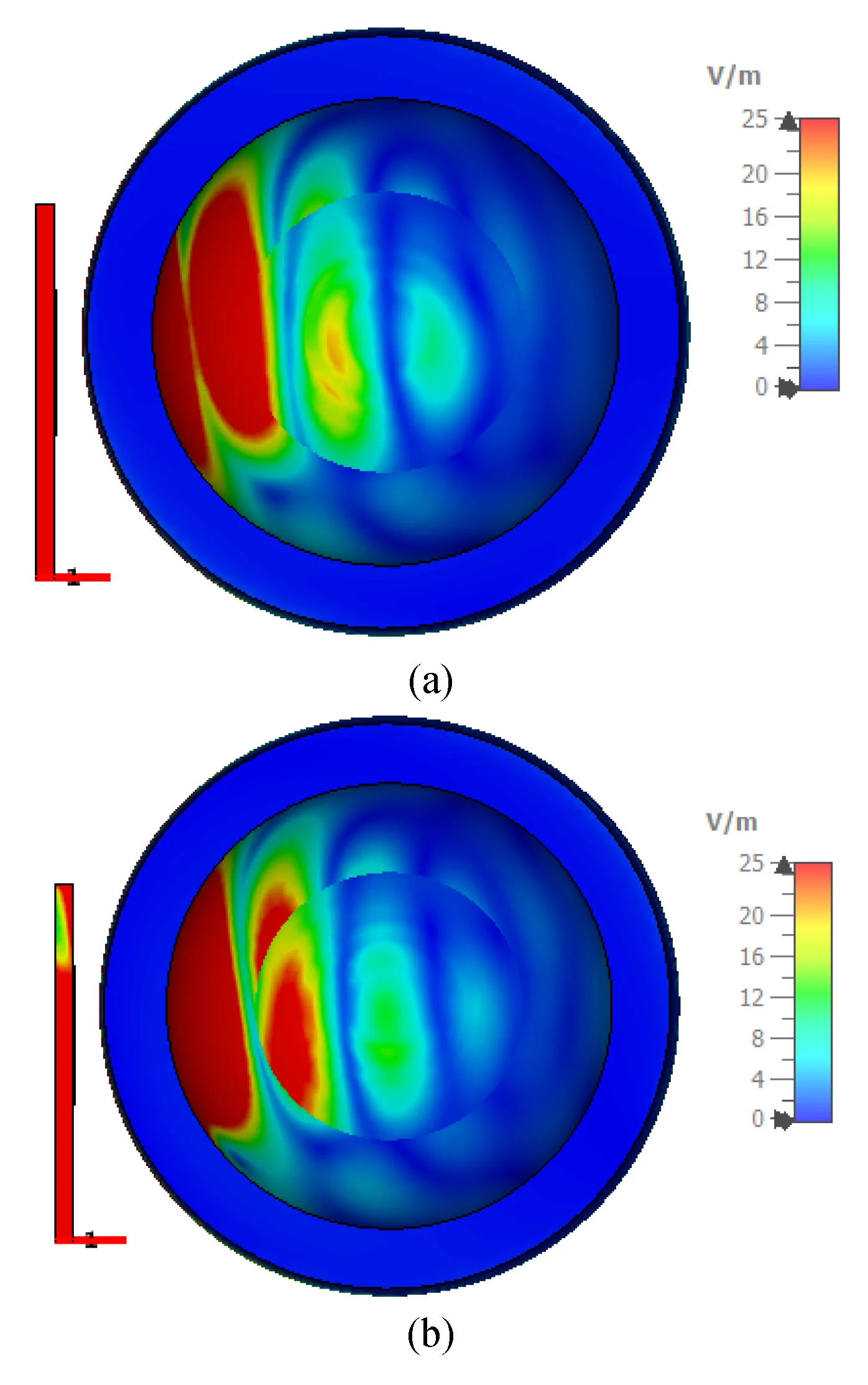
Simulated electromagnetic response of the proposed antenna: (a) electric-field distribution inside the phantom-loaded model at 0°, (b) electric-field distribution inside the phantom-loaded model at 90°.

Figures 2 and 3 should be evaluated together because they show how the electric field is formed around the unloaded antenna and how this field interacts with the phantom. Figure 2 presents the simulated electric-field magnitude of the antenna at 3.8 GHz without the phantom. The 0°, 90°, and 145° planes show different cross-sectional views of the same electromagnetic state and do not represent different operating conditions.

As shown in Fig. 2(a-c), the electric field is not distributed uniformly over the antenna surface. The highest field values are located close to the resonator edges and around the curved sections defined by R1, R2, and R3. These regions contain strong fringing fields that extend from the resonator surface into the surrounding space. The field becomes weaker as the distance from the antenna surface increases. Therefore, the regions close to the curved boundaries form the main sensing zone of the proposed structure.

Figure 3 shows the electric-field distribution after the multilayer phantom is placed above the antenna. In both observation planes, the field crosses the antenna-phantom interface and penetrates into the phantom. The strongest field remains close to the antenna-facing surface and gradually decreases with increasing distance from the resonator. This result shows that the phantom is located within the reactive near-field region of the antenna and can directly affect the electromagnetic energy stored around the resonator.

**Figure 4:**
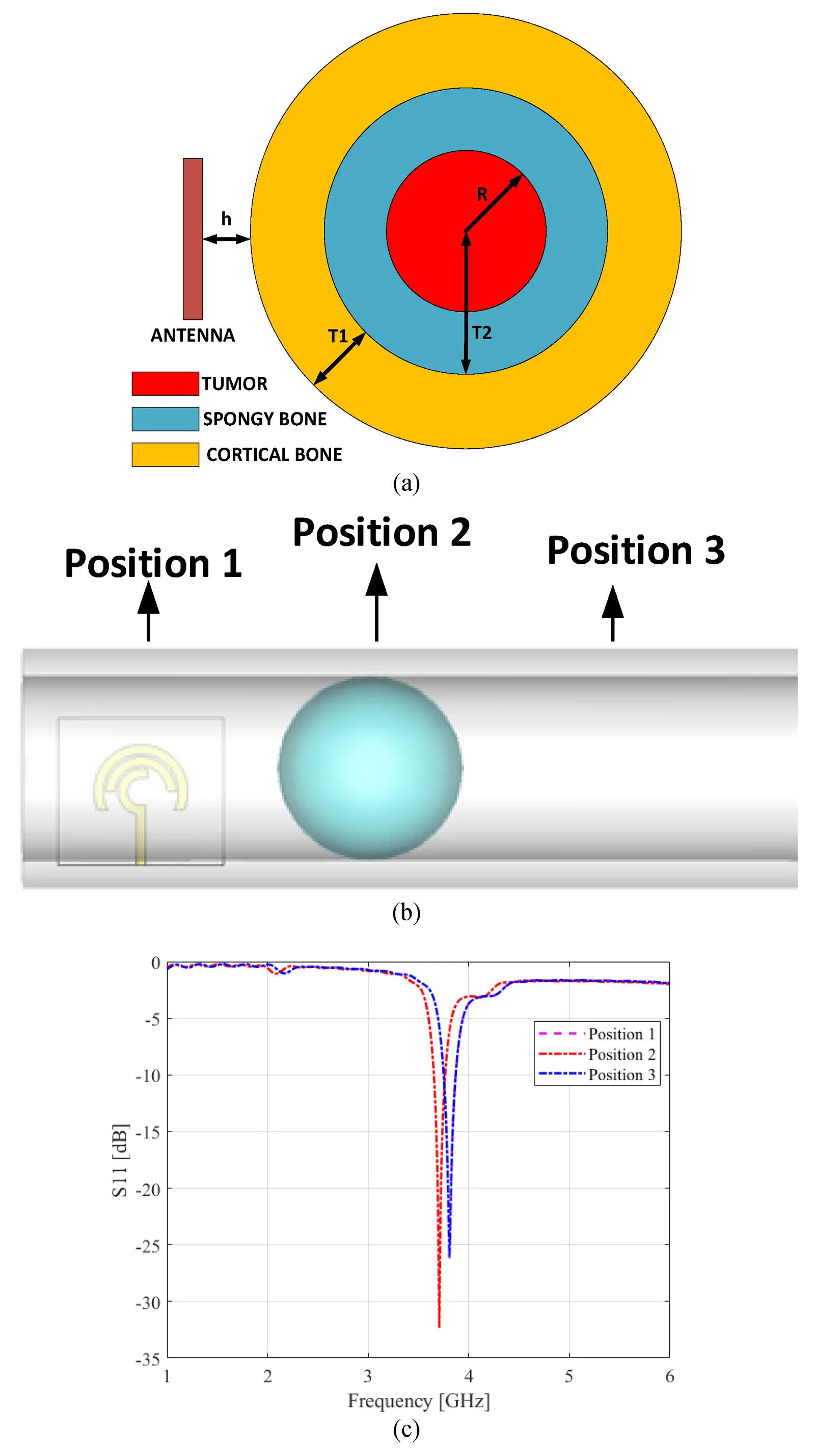
Multilayer bone phantom model and antenna measurement positions: (a) cross-sectional phantom geometry, (b) CST-based sensing configuration with healthy and tumor-loaded positions and (c) CST-based S_11_ measurements

The link between the field distribution and the simulated S_11_ response can be explained through dielectric loading. When the phantom overlaps with the concentrated electric-field region, its relative permittivity changes the effective permittivity experienced by the resonator. A material with a higher real permittivity increases the electric energy stored in the sensing region and generally shifts the resonance toward a lower frequency. At the same time, a higher imaginary permittivity introduces additional dielectric loss and can change the depth and width of the S_11_ notch. Therefore, the phantom-loaded electric-field behavior shown in Fig. 3 provides the physical explanation for the change in the simulated S_11_ response shown in Fig. 4(c).

The comparison of Figs. 2 and 3 confirms that the proposed antenna has sufficient field overlap with the phantom. It also shows that a tumor-like region placed inside this field-sensitive volume can disturb the local effective permittivity and produce a measurable change in resonance frequency and S_11_ magnitude.

### Bone Phantom and Tumor Region Preparation

To evaluate the sensing capability of the proposed microstrip resonator antenna under controlled conditions, a multilayer bone phantom and a separate tumor-region phantom were prepared using tissue-mimicking formulations. The phantom model consisted of three regions: cortical bone, marrow, and tumor. These layers were designed to reproduce the dielectric contrast expected between healthy bone tissues and malignant regions in the microwave frequency range.

The cortical bone and marrow phantoms were prepared using wheat flour, deionized water, dextrose, and olive oil with different proportions. Wheat flour acted as the structural matrix, olive oil reduced the effective permittivity by representing the lipid-rich composition of bone tissues, and dextrose contributed to adjusting the dielectric loss. The marrow formulation contained higher water and dextrose contents to represent its relatively higher hydration level compared with cortical bone 17.

The tumor-region phantom was prepared separately using deionized water, sunflower oil, and gelatin. Its water-dominant composition was selected to provide higher ε′ and ε″ values than the bone phantom, consistent with the elevated water content and ionic nature of malignant tissue. This dielectric contrast enables measurable perturbation of the antenna response when the tumor region is located within the near-field sensing zone. The complete formulations are listed in Table 2.

The phantom assembly was also modeled in CST to support the simulation of phantom-loaded antenna behavior prior to fabrication. The CST phantom model, shown in Fig. 4, represents the layered geometry and relative dimensions of the bone phantom as used in the experimental configuration.

## Results and Discussion

### Fabrication of the Antenna Prototype

After simulation-based optimization, the microstrip resonator antenna was fabricated on an FR4 substrate using a standard printed circuit board etching process. The fabricated prototype follows the geometrical parameters given in Table 1. The conductive resonator pattern was formed on one side of the FR4 substrate, while the ground plane was placed on the opposite side. A standard SMA connector was soldered to the feed point for connection to the measurement system.

**Figure 5:**
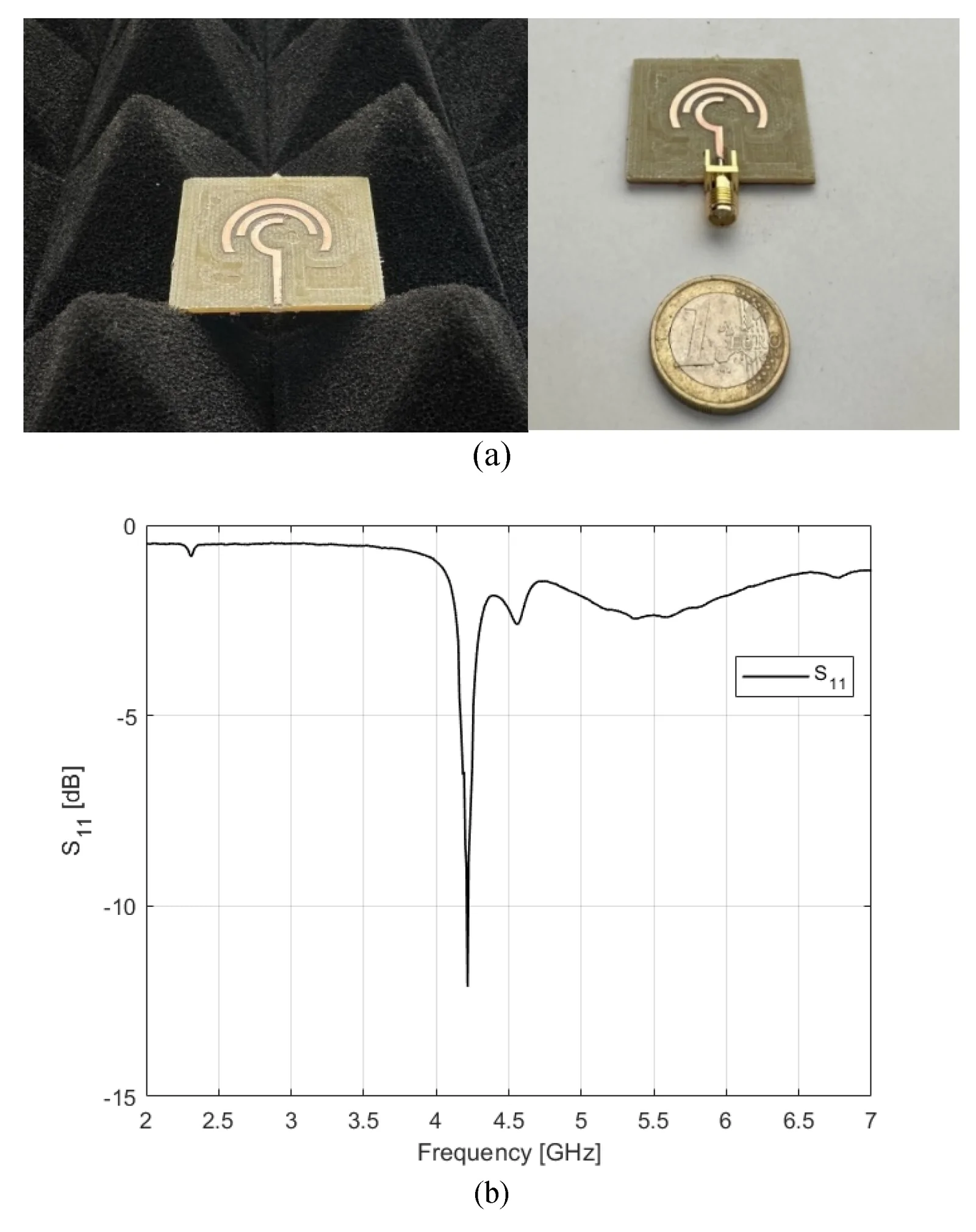
(a) Fabricated prototype of the proposed microstrip resonator antenna on FR4 substrate (εr = 4.6, h = 1.56 mm), (b) S_11_ result in CST.

The fabricated antenna prototype is shown in Fig. 5(a). The prototype has a compact and planar geometry, which is suitable for near-field phantom sensing measurements. The simulated S_11_ response obtained in CST is shown in Fig. 5(b), confirming the expected resonant behavior of the designed antenna before experimental testing. The use of FR4 also supports a simple and low-cost fabrication route using standard laboratory facilities.

### Dielectric Characterization of Phantom Materials

Dielectric characterization of the phantom materials was carried out because the sensing mechanism depends on the dielectric contrast between the bone phantom and the tumor-like region. The complex relative permittivity of both materials was measured using a VNA-based open-ended coaxial probe system. The measured data were expressed as the real part of permittivity (ε′), which is related to electric energy storage, and the imaginary part of permittivity (ε″), which is related to dielectric loss.

**Figure 6:**
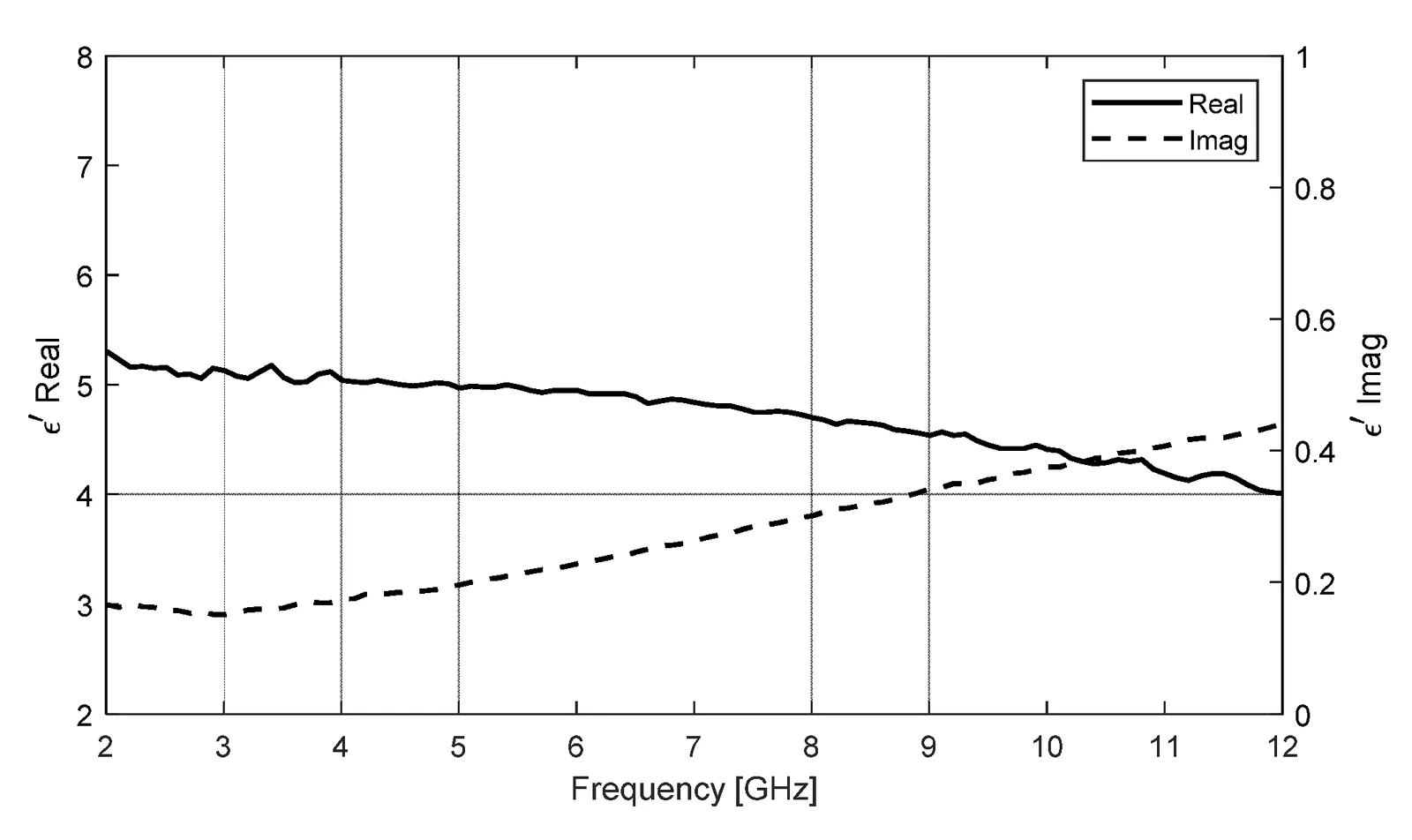
Measured frequency-dependent real (ε′) and imaginary (ε″) parts of the relative permittivity of the fabricated bone phantom.

**Figure 7:**
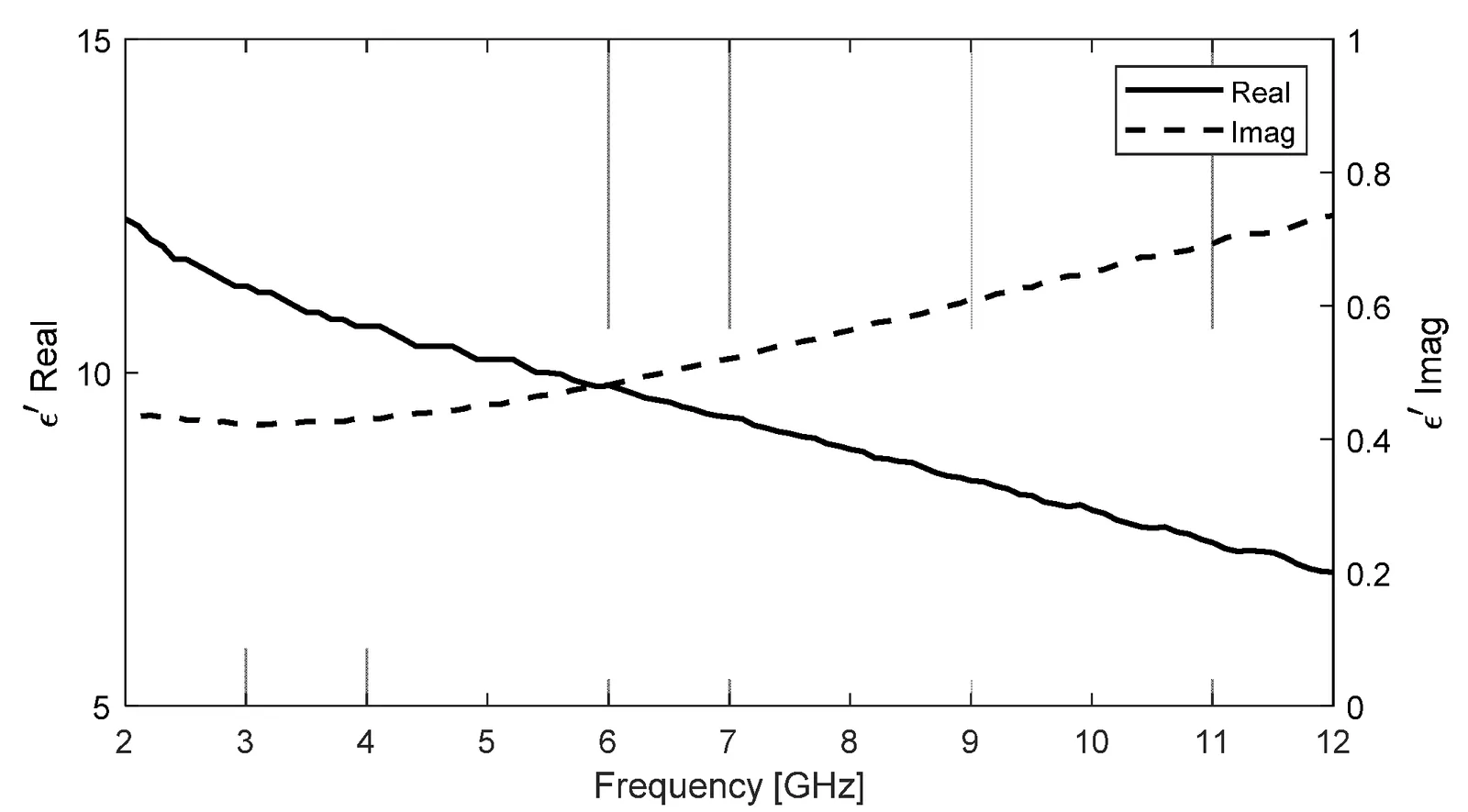
Measured dielectric properties of the tumor-region showing the frequency-dependent real (ε′) and imaginary (ε″) components of relative permittivity.

The measured ε′ and ε″ curves of the fabricated bone phantom and tumor-like region are shown in Fig. 6 and Fig. 7, respectively. The bone phantom showed lower dielectric values over the measured frequency range. Its ε′ decreased from approximately 5.2 to 4.1, while ε″ increased from approximately 0.17 to 0.45 between 2 and 12 GHz. This behavior is consistent with the oil-containing and relatively low-water composition of the bone phantom.

In contrast, the tumor-like region showed clearly higher dielectric values. Its ε′ decreased from approximately 12.2 to 7.0, while ε″ increased from approximately 0.42 to 0.75 over the same frequency range. This higher dielectric response is mainly related to the water-rich gelatin-based formulation of the tumor-like material. The observed dielectric contrast between the two materials supports the expected near-field loading mechanism of the microstrip resonator antenna. Therefore, when the tumor-like region is placed within the antenna sensing zone, it can change the effective permittivity around the resonator and produce a measurable change in the S_11_ response.

### Resonance Frequency Shift Mechanism

**Figure 8:**
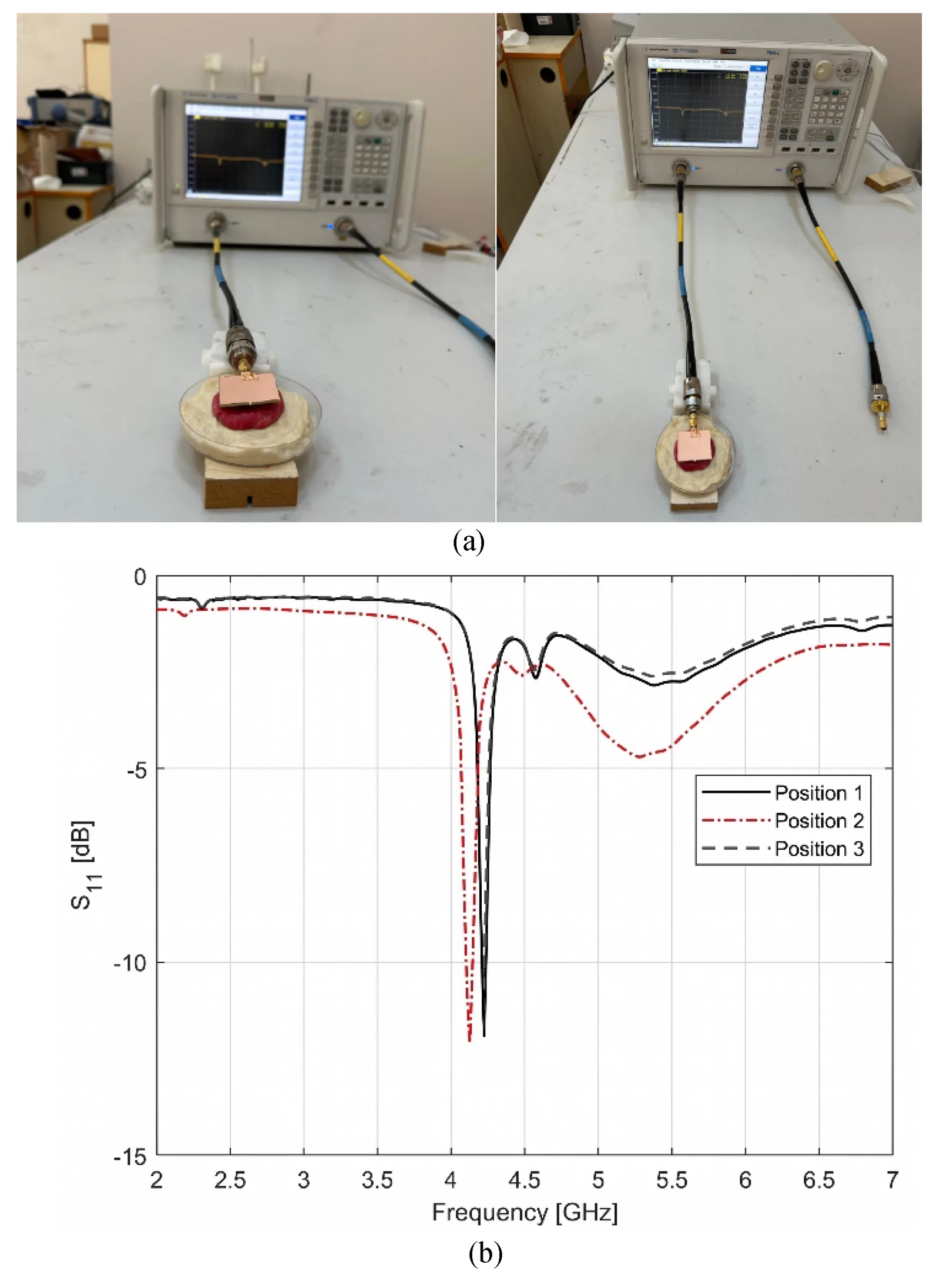
Measured S₁₁ responses of the proposed antenna for healthy (Position 1, position 3) and tumor-over-phantom (position 2) configurations.

The measured reflection coefficient (S_11_) responses of the fabricated microstrip resonator antenna at three phantom positions are shown in Fig. 8. Positions 1 and 3 represent healthy phantom regions, while Position 2 represents the tumor-over-phantom configuration. The healthy positions show similar resonance behavior, with the main resonance notch located around the 4.2-4.3 GHz range. This similarity indicates that the antenna response is reasonably consistent when measured over different healthy regions of the phantom.

When the antenna is positioned over the tumor-like region at Position 2, the measured S_11_ response changes clearly compared with the healthy positions. The main resonance shifts to a lower frequency, and the depth and shape of the S_11_ notch are modified. This behavior is consistent with the near-field dielectric loading mechanism. The tumor-like region has higher ε′ and ε″ values than the surrounding bone phantom, which changes the effective permittivity around the antenna fringing fields and adds dielectric loss to the sensing region.

The observed difference between the healthy and tumor-over-phantom responses shows that the fabricated microstrip resonator antenna can detect the dielectric contrast introduced by the tumor-like region under controlled phantom conditions. Therefore, the measured S_11_ response supports the preliminary sensing capability of the proposed antenna for phantom-based bone tumor sensing.

The resonance frequency shift between the healthy and tumor-over-phantom configurations was analyzed using the resonance frequencies extracted from the measured S_11_ curves. Positions 1 and 3 were used as healthy reference locations, while Position 2 corresponded to the tumor-loaded region. The average resonance frequency of the two healthy positions was defined as the reference frequency for bone cancer diagnostics.

For the resonance-shift analysis, the healthy reference frequency was defined as the average of the two healthy phantom measurements, namely Position 1 and Position 3:


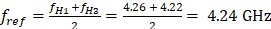
(1)

The left-right healthy variability was calculated as the absolute difference between the two healthy measurements:


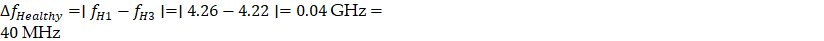
 (2)

In the presence of a tumor inclusion, the resonance frequency shifts to:


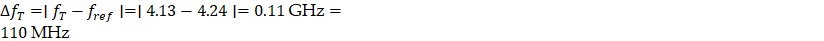
 (3)

The resulting sensitivity ratio (SR), defined as the ratio of tumor-induced shift to healthy variability, is calculated as:


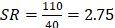
(4)

In addition to frequency displacement, the tumor-loaded configuration exhibits a 4.47 dB increase in the magnitude of the reflection coefficient, calculated as:


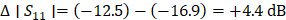
(5)

This result indicates that the tumor-induced frequency shift is approximately 2.75 times larger than the variation observed between the two healthy phantom positions. Therefore, the measured shift can be attributed to the dielectric perturbation introduced by the tumor-like region rather than normal positional variation.

Physically, this shift occurs because the resonance frequency of a microstrip resonator depends on the effective permittivity experienced by its fringing electric fields. When the antenna is placed over the tumor-like region, the higher dielectric constant of the tumor phantom increases the local effective permittivity, resulting in a downward shift of the resonance frequency. In addition, the higher dielectric loss of the tumor phantom modifies the depth and shape of the S_11_ notch. The measured 4.47 dB change in S_11_ magnitude further confirms that the tumor-loaded region alters the impedance-matching condition of the antenna. Overall, the frequency shift and S_11_ magnitude variation demonstrate that the proposed antenna can detect the dielectric contrast between healthy and tumor-like phantom regions.

Table 3 summarizes recent antenna-based studies for cancer detection and imaging. Previous works mainly focused on lung, brain, and breast cancer using microstrip patch, Vivaldi, or wearable antenna structures over different microwave frequency ranges. Compared with these studies, the present work focuses specifically on bone cancer sensing using a compact FR4-based microstrip resonator antenna operating around 4.0-4.5 GHz. This comparison highlights the potential of the proposed low-cost and compact antenna as a non-ionizing sensing platform for phantom-based bone tumor detection.

## Conclusion

This study presented a proof-of-concept microwave sensing approach for phantom-based bone tumor sensing using a compact microstrip resonator antenna fabricated on an FR4 substrate. The antenna was designed in CST Microwave Studio, fabricated, and experimentally tested through S_11_ measurements. A multilayer bone phantom with cortical bone- and marrow-mimicking regions was prepared together with a water-rich gelatin-based tumor-like region. Dielectric measurements confirmed a clear contrast between the bone phantom and the tumor-like material in both ε′ and ε″.

The measured S_11_ results showed that the tumor-over-phantom configuration produced a clear response change compared with the healthy phantom positions. The tumor-like region caused a downward resonance shift of 110 MHz relative to the healthy reference, which was 2.75 times larger than the variation between the two healthy positions. A 4.47 dB change in S_11_ magnitude was also observed. These results support the near-field dielectric loading mechanism, where the higher permittivity and loss of the tumor-like material change the antenna response.

However, the results should be interpreted as a controlled phantom-based sensing study, not as a clinical diagnostic result. The phantoms used in this study cannot fully represent the heterogeneity, anisotropy, vascularization, and patient-specific variability of real bone and tumor tissues. In addition, the effects of tumor size, depth, geometry, and repeated-measurement variability were not systematically studied. Future work should include more realistic multilayer phantoms, different tumor sizes and depths, improved resonator sensitivity, and multi-position or array-based measurements. Data-driven analysis of S_11_ features may also help separate healthy and tumor-like responses under more variable measurement conditions.

## Figures and Tables

**Table 1:** Geometrical parameters of the proposed microstrip resonator antenna

Parameter	Value (mm)	Parameter	Value (mm)
L1	11.2	W1	2.0
L2	32.0	W2	36.0
R1	3.0	R3	10.2
R2	7.6	Substrate	FR4 (εr = 4.6, h = 1.56 mm)

**Table 2:** Formulation Recipes for the Multilayer Bone Phantom and Tumor Region

Component	Cortical Bone	Marrow	Tumor Region
Wheat Flour	60 g	60 g	-
Deionized Water	25 mL	40 mL	25.5 mL
Dextrose	7.5 g	15.2 g	-
Olive Oil	35 mL	30 mL	-
Sunflower Oil	-	-	1 mL
Gelatin	-	-	5 g

**Table 3:** Studies on cancer detection and imaging

Ref	Antenna type	Cancer type	*f*	Size (mm³)	Purpose
17	Microstrip patch- Rogers 3003	Bone cancer	7-10 GHz	36×30×4.85	Detection
21	Microstrip patch- FR4	Lung cancer	2.5-7.5 GHz	60×40×1.6	Imaging
22	Microstrip patch- FR4	Lung cancer	1-5 GHz	55×65×1.6	Imaging
23	Vivaldi- FR4	Brain cancer	2-8 GHz	55×65×1.6	Detection
24	wearable antenna- Cotton	Breast cancer	1.6-10 GHz		Breast-cancer detection; Smart Bra; ML-assisted
25	Zelt patch- Felt	Breast cancer	8-12 GHz		Breast-cancer detection; SAR compliant
This work	microstrip resonator antenna- FR4	Bone cancer	4.0-.4.5 GHz	32x36x1.56	Sensing

## References

[2] Hosseini H, Heydari S, Hushmandi K, Daneshi S, Raesi R (2025). Bone tumors: a systematic review of prevalence, risk determinants, and survival patterns. Vol. 25, BMC Cancer. BioMed Central Ltd.

[3] Brodowicz T, Hadji P, Niepel D, Diel I (2017). Early identification and intervention matters: A comprehensive review of current evidence and recommendations for the monitoring of bone health in patients with cancer. Vol. 61, Cancer Treatment Reviews. W.B. Saunders Ltd.

[4] Akram Z, Faraz M, Fatima U (2025). Role of CT Scan and MRI in Cancer Diagnosis: A Comparative Review of Imaging Modalities for Precision Medicine. Journal of Cancer and Tumor International.

[5] Heck L, Herzen J (2020). Recent advances in X-ray imaging of breast tissue: From two- to three-dimensional imaging. Vol. 79, Physica Medica. Associazione Italiana di Fisica Medica.

[6] Bainbridge H, Dubec MJ, Wetscherek A (2026). Magnetic resonance imaging thoracic organ-at-risk atlas for radiation oncology. Phys Imaging Radiat Oncol.

[7] Du J, Bydder GM (2013). Qualitative and quantitative ultrashort-TE MRI of cortical bone. Vol. 26, NMR in Biomedicine.

[8] Amin B, Elahi MA, Shahzad A, Porter E, McDermott B, O'Halloran M (2019). Dielectric properties of bones for the monitoring of osteoporosis. Med Biol Eng Comput [Internet].

[9] Amin B, Mehboob A, Dunne E (2025). Dielectric Models of Normal and Diseased Human Trabecular Bones at Microwave Frequencies. IEEE Open Journal of Antennas and Propagation [Internet].

[10] Bai J, Guo H, Lai X (2025). An Electronically Controlled Resonant Microwave Sensor Array for Skin Tumor Detection. IEEE Access.

[11] Meaney PM, Zhou T, Goodwin D, Golnabi A, Attardo EA, Paulsen KD (2012). Bone dielectric property variation as a function of mineralization at microwave frequencies. Int J Biomed Imaging. 2012.

[12] Islam ZU, Bermak A, Wang B (2024). A Review of Microstrip Patch Antenna-Based Passive Sensors. Vol. 24, Sensors. Multidisciplinary Digital Publishing Institute (MDPI).

[13] Khalesi B, Sohani B, Ghavami N, Ghavami M, Dudley S, Tiberi G (2019). A phantom investigation to quantify huygens principle based microwave imaging for bone lesion detection. Electronics (Switzerland).

[14] Mezaal YS (2026). Investigation of Printed Slot Antenna for Non-Invasive Glucose Sensing Using FR4 Substrate Material. Micromachines (Basel).

[15] Särestöniemi M, Singh D, Dessai R, Heredia C, Myllymäki S, Myllylä T (2024). Realistic 3D Phantoms for Validation of Microwave Sensing in Health Monitoring Applications. Sensors.

[16] Abedi S, Joachimowicz N, Phillips N, Roussel H (2021). A simulation-based methodology of developing 3D printed anthropomorphic phantoms for microwave imaging systems. Diagnostics.

[17] Akdogan V, Teksen FA, Haxha S, Karaaslan M (2025). A New Perspective for Early Detection of Bone Tumour: Metamaterial-Based Antenna Solution. Plasmonics.

[18] Dey P, Vaideanu A, Mosca S (2022). Surface enhanced deep Raman detection of cancer tumour through 71 mm of heterogeneous tissue. Nanotheranostics.

[19] Dinari A, Ahmad HA, Oh S, Kim YH, Yoon J (2025). Advanced Detection of Pancreatic Cancer Circulating Tumor Cells Using Biomarkers and Magnetic Particle Spectroscopy. Nanotheranostics.

[20] Li Z, Liu X, Zhang W, Zhuang X (2020). Electrostatic reaction for the detection of circulating tumor cells as a potential diagnostic biomarker for metastasis in solid tumor. Nanotheranostics.

[21] Alhawari ARH (2018). Lung Tumour Detection Using Ultra-Wideband Microwave Imaging Approach. Journal of Fundamental Applied Science [Internet].

[22] Alamro W, Seet BC, Wang L, Parthiban P (2023). Early-Stage Lung Tumor Detection Based on Super-Wideband Microwave Reflectometry. Electronics (Switzerland).

[23] Reem Alagee E, Assalem A (2020). Brain Cancer Detection Using U-Shaped Slot VIVALDI Antenna and Confocal Radar Based Microwave Imaging Algorithm. Technology, and Sciences (ASRJETS) American Scientific Research Journal for Engineering [Internet].

[24] Asha Banu S.M, Meena Alias Jeyanthi K (2022). Textile based antenna design for breast cancer detection. In: Materials Today: Proceedings. Elsevier Ltd.

[25] Srinivasan D, Gopalakrishnan M (2019). Breast Cancer Detection Using Adaptable Textile Antenna Design. J Med Syst.

